# Evaluation of the Efficacy and Safety of CollaSel PRO^®^ Type I and Type III Hydrolyzed Collagen Peptides in the Treatment of Osteoarthritis: A Double-Blind, Placebo-Controlled, Randomized Clinical Trial

**DOI:** 10.3390/jcm14113655

**Published:** 2025-05-23

**Authors:** Devrim Demir-Dora, Serpil Tuna, Emel Dogan Kurtoglu, Savas Gursoy, Nilufer Balci, Selim Tezman, Aydin Erenmemisoglu

**Affiliations:** 1 Department of Medical Pharmacology, Faculty of Medicine, Akdeniz University, 07070 Antalya, Turkey; devrimdemirdora@akdeniz.edu.tr; 2 Department of Gene and Cell Therapy, Faculty of Medicine, Akdeniz University, 07070 Antalya, Turkey; 3 Department of Physical Therapy and Rehabilitation, Faculty of Medicine, Akdeniz University, 07070 Antalya, Turkey; serpiltuna@akdeniz.edu.tr (S.T.);; 4 Novagenix Bioanalytical Drug R&D Centre, 06750 Ankara, Turkey; edogan@novagenix.com; 5 Department of Physical Therapy and Rehabilitation, Faculty of Medicine, Gaziantep University, 27310 Gaziantep, Turkey; gursoysavas34@gmail.com; 6 Sel Sanayi Urunleri Ticaret ve Pazarlama AS, 34440 Istanbul, Turkey; stezman@tezmanholding.com; 7 Alpan Farma R&D Ltd., 38039 Kayseri, Turkey

**Keywords:** osteoarthritis, hydrolyzed collagen peptide, type I collagen, type III collagen, WOMAC, AOFAS-AHFS

## Abstract

**Objectives**: This study aims to evaluate the efficacy and safety of oral supplementation with hydrolyzed collagen peptides (HCP) type I and type III (CollaSel PRO^®^) in reducing joint pain and stiffness and improving physical function in patients with osteoarthritis (OA). **Methods**: In this randomized, double-blind, placebo-controlled trial (ClinicalTrials.gov Identifier: NCT05369780; approved by the Institutional Review Board), 160 adult patients with OA (mean age 52.4 ± 4.3 years; 111 females, 49 males) were randomly assigned to receive either 10 g/day of CollaSel PRO^®^ (n = 80) or placebo (maltodextrin, n = 80) for 8 weeks. The Western Ontario and McMaster Universities Osteoarthritis Index (WOMAC) was used to evaluate knee and hip joints, and the American Orthopaedic Foot and Ankle Society Ankle-Hindfoot Scale (AOFAS-AHFS) was used for ankle joints. Assessments were conducted at baseline and at weeks 1, 4, and 8. **Results**: In the CollaSel PRO^®^ group, a significant decrease in WOMAC scores (mean ± SD 50.5 ± 17.0, 40.7 ± 16.9 and 33.8 ± 16.5 vs. 53.7 ± 16.9, respectively, *p* < 0.001 for each) and significant increase in AOFAS-AHFS scores (64.2 ± 13.1, 73.6 ± 12.3 and 80.8 ± 9.9 vs. 61.8 ± 13.9, respectively, *p* < 0.001 for each) were noted at weeks 1, 4, and 8 compared to the baseline scores. WOMAC scores were significantly lower in comparing HCP and placebo, and AOFAS-AHFS scores were higher in the HCP group compared to the placebo group at weeks 4 and 8 (all *p* < 0.001). **Conclusions**: CollaSel PRO^®^ appears to be a safe and effective option for the symptomatic management of osteoarthritis. It significantly improved joint pain, stiffness, and physical function in multiple joints. These improvements are not only statistically significant but also clinically meaningful, supporting the need for longer-term evaluation.

## 1. Introduction

Osteoarthritis (OA) is the most prevalent joint disease, mainly defined by the degeneration of articular cartilage. OA is more prevalent in older patients, and general life expectancy is constantly increasing; thus, the burden of OA is on the rise [1,2]. OA primarily impacts weight-bearing joints like the knee and hip, causing chronic joint pain, stiffness, and decreased function [3,4]. Advanced age, female gender, obesity, occupational overuse, repetitive trauma, musculoskeletal injuries, and history of professional sports activity are risk factors contributing to the development of OA [5].

Current therapeutic approaches to OA, such as paracetamol, nonsteroidal anti-inflammatory drugs (NSAIDs), intra-articular corticosteroids, and physical therapy, generally focus on symptomatic treatment [6,7]. However, since these conventional treatments are associated with safety concerns and limited short-term therapeutic efficacy, the need for safer disease-modifying alternatives offering chondroprotective or regenerative capacity is considered a significant gap in OA treatment [7,8]. Therefore, there has been increased interest in nutraceuticals and dietary supplements with potential chondroprotective or regenerative effects. The potential beneficial effects of various nutraceuticals and dietary supplements (e.g., glucosamine, chondroitin sulfate, hyaluronic acid, vitamin D, and collagen derivatives) in improving joint health have been demonstrated in several clinical studies in OA patients, with collagen derivatives, in particular, considered to provide more significant benefits [9,10,11,12,13,14]. Various forms of collagen supplements exist, including undenatured collagen, gelatin, and hydrolyzed collagen. Hydrolyzed collagen, in particular, is considered the most suitable candidate for disease-modifying therapy due to its high bioavailability, broad biological effects, and potential to support cartilage repair and regeneration [9,10,11,14]. Hydrolyzed collagen, obtained by denaturation of native collagen, consists of segmented proteins with low molecular weight enabling their distribution across several tissues quickly after digestion. Previous studies have shown that the bioactive peptides of oral collagens have relatively fast absorption due to their low molecular weight. They are digested and metabolized to dipeptides and tripeptides in the gastrointestinal tract and thereafter transported through the bloodstream [15].

Glycine, proline, and hydroxyproline make up approximately 50% of the total amino acids of collagen [16]. Hydroxyproline is a collagen-specific amino acid, and the main peptide found in plasma after ingestion of HC is proline-hydroxyproline, which functions as a trigger for collagen synthesis and extracellular matrix reorganization. The mechanism of action of HC is a chemotactic stimulus for fibroblasts with attraction of cells to repair tissue damage [17].

However, the literature on collagen supplementation in OA remains inconsistent. Most existing studies focus on knee OA, while evidence for other joints such as the hip and ankle is limited [9,10,11,12,14,18]. Despite the lack of strong guideline recommendations, these collagen-based products are heavily marketed and widely used by patients [9,10,12,14]. Moreover, most of these clinical studies use combination products containing additional ingredients like vitamins, antioxidants, coenzyme Q10, hyaluronic acid, and chondroitin sulfate. These may exert synergistic effects and confound the true efficacy of collagen.

To our knowledge, there are no randomized controlled trials in the literature evaluating the effects of pure type I and III hydrolyzed collagen peptide formulations without additional compounds in patients with knee, hip, and ankle OA.

This placebo-controlled, randomized clinical trial aims to evaluate the efficacy and safety of 8-week oral supplementation of pure type I and type III hydrolyzed collagen peptides (CollaSel PRO^®^), on pain, stiffness, and physical function in patients with OA. The novelty of this study lies in its use of pure type I and type III hydrolyzed collagen peptides without any additional excipients and its inclusion of joints beyond the knee, namely the hip and ankle.

## 2. Patients and Methods

### 2.1. Manufacturing and Quality Evaluation of Collagen

Specific requirements for collagen peptides for human use are laid down in Regulation (EC) No 853/2004 of the European Parliament and of the Council, in which there are requirements about raw materials, transport and storage conditions of raw materials, collagen manufacturing process, and collagen quality. Collagen has been manufactured according to the ‘Gelatine Manufacturers of Europe Collagen Peptides Monograph’ for European Union (EU). The physical, chemical, and microbiological quality requirements of collagen were evaluated according to this monograph. Bovine hide was used as a collagen source, and hydrolyzed collagen peptides type I and type III were extracted and purified from the bovine hide. All characterizations have been done strictly following the necessary standards. CollaSel PRO^®^ hydrolyzed collagen peptide was approximately 2000 Daltons, and the amino acid composition was determined as alanine, arginine, aspartic acid, glutamic acid, glycine, histidine, hydroxyproline, isoleucine, leucine, lysine, ornithine, phenylalanine, proline, serine, threonine, tyrosine, valine, and methionine. The color, clarity, moisture, ash, pH, conductivity, viscosity, SO_2_, and H_2_O_2_ conformed to the specifications. The total aerobic microbial count, coliform bacteria count, and Escherichia coli count were within the specifications. The product was harmonious with all referred quality standards in the above-mentioned monograph. Before submission to EC and MOH, all evaluations were documented in detail.

### 2.2. Study Design

A total of 160 patients with knee, hip, and ankle OA who applied to two University Hospital’s Physical Medicine and Rehabilitation clinics were included in this study. The patients had a mean age of 52.4 ± 4.3 years, and 69.4% were female.

The patients were randomly assigned in a 1:1 ratio using a computer-generated randomization table into two groups by an independent researcher not involved in the study procedures. Patients in the test group (n = 80) received a single daily oral dose of 10 g CollaSel PRO^®^ hydrolyzed collagen peptide for 8 weeks, while patients in the placebo group (n = 80) received a single daily oral dose of 10 g maltodextrin for 8 weeks. Both the patients and outcome assessors were blinded to group allocation. The study products were provided in identical packaging and labeled with code numbers by a third party to ensure allocation concealment. The data analyst remained blinded until the statistical analysis was completed.

**Figure 1:**
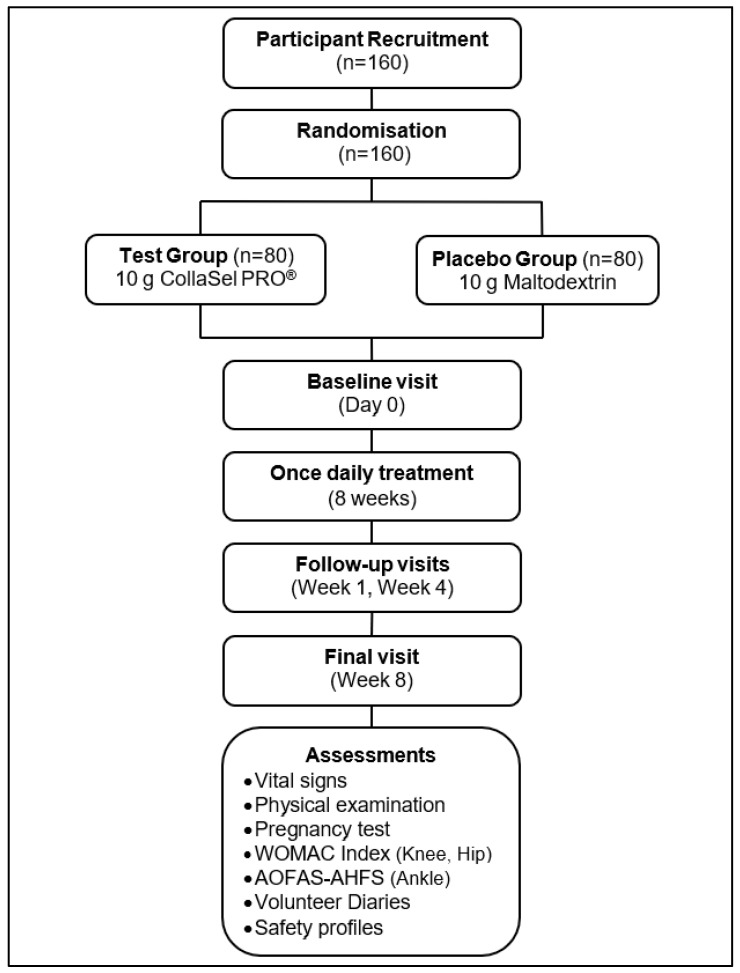
Study flowchart.

The patients were evaluated at baseline and at weeks 1, 4, and 8. This study is a multiple-dose, randomized, double-blind, placebo-controlled, parallel-design clinical trial conducted over 5 months between 24 May 2022 and 22 September 2022 (ClinicalTrials.gov Identifier: NCT05369780) (Figure 1).

### 2.3. Inclusion Criteria

- Patients with OA aged between 45 and 60 years,
- Patients diagnosed with knee or hip OA who were not drug-naïve to OA and related conditions,
- Patients with normal blood pressure (systolic: 110–140 mmHg, diastolic: 60–90 mmHg) and heart rate (50–100 bpm) measured after 5 min of rest during the screening visit,
- Patients who communicated adequately with the investigator, complied with the study requirements, and agreed to participate.

### 2.4. Exclusion Criteria

- Patients with an atopic constitution or asthma and/or a known allergy to collagen products or any of the excipients of the product,
- Patients with hereditary conditions such as galactose intolerance, Lapp lactase deficiency, or glucose–galactose malabsorption,
- Patients with diabetes mellitus,
- Patients with serious or life-threatening conditions affecting the cardiovascular, neurological, hematological, hepatic, gastrointestinal, renal, pulmonary, endocrinological, metabolic, or psychiatric systems,
- Patients with any porphyria,
- Patients with swallowing difficulties, malabsorption, or a history of gastrointestinal surgery (except appendectomy or herniotomy),
- Patients with active rheumatoid arthritis or any other inflammatory arthritic condition deemed inappropriate by the researchers,
- Patients with a history of planned or prior joint-related reconstructive surgery,
- Patients who have used oral retinoids or oral steroids within six months before the start of the study,
- Patients currently or regularly taking NSAIDs, contraceptives, hormones, obesity drugs, absorption inhibitors, or antidepressants,
- Patients deemed unlikely to comply with the study procedures or complete the study based on the investigator’s judgment.

Written informed consent was obtained from each subject. This study was conducted by the ethical principles stated in the ‘Declaration of Helsinki’ and approved by the Gaziantep University Clinical Research Ethics Committee (Date of Approval: 29 December 2021, Approval No: 2021/05) and the Republic of Turkey Ministry of Health Turkish Medicines and Medical Devices Agency (Date of Approval: 12 April 2022, Letter No: E-66175679-514.11.01-730666).

### 2.5. Assessments

At the baseline visit, data on patient demographics (age, gender) and medical history were recorded, along with explanations regarding filling out the volunteers’ diary forms and using the study product.

Joint status was evaluated with the Western Ontario and McMaster Universities Arthritis Index (WOMAC) for the knee and hip joints and with the American Orthopedic Foot and Ankle Society Ankle-Hindfoot Scale (AOFAS-AHFS) for the ankle joint at each study visit (baseline and weeks 1, 4, 8 of treatment) in the test product and placebo groups. The contribution of hydrolyzed collagen peptide to joint pain, stiffness, and physical function was investigated against a placebo.

Data on vital signs (body temperature, blood pressure, heart rate, respiratory rate), physical examination, and pregnancy test (urine test for those with child-bearing potential) were also recorded at each study visit. Data on the volunteer’s self-completed diary forms were recorded during the treatment period visits (weeks 1, 4, and 8). In contrast, the volunteers’ compliance with the drug administration study and the safety data on adverse events (AEs, reported by the volunteer or observed by the investigator) were recorded during the treatment period visits (weeks 1, 4, and 8) (Figure 1).

### 2.6. Western Ontario and McMaster Universities Arthritis Index (WOMAC)

The WOMAC is a self-administered 24-item questionnaire widely used to evaluate hip and knee OA. Each item of the three subscales, including pain (5 items), stiffness (2 items), and physical function (17 items), is scored on a scale of 0–4 as none (score 0), mild (score 1), moderate (score 2), severe (score 3), and extreme (score 4). The overall WOMAC score is determined by summing the scores from its three subscales, with higher values indicating increased levels of pain, stiffness, and functional limitations [19].The validity and reliability analysis of the Turkish version of WOMAC was performed by Basaran et al. in 2010 [20].

### 2.7. American Orthopedic Foot and Ankle Society Ankle-Hindfoot Scale (AOFAS-AHFS)

AOFAS-AHFS is a standardized evaluation of the clinical status of the ankle-hindfoot that incorporates both subjective and objective information, including patient-reported pain (40 points) and function (activity limitations, support requirements, 50 points) assessed together by patient and physician, as well as physician-based assessment of alignment (10 points). The total score ranges from 0 (indicating severe pain and impairment) to 100 (no impairment) points [21]. Analay Akbaba et al. performed a validity and reliability analysis of the Turkish version of AOFAS-AHFS in 2016 [22].

### 2.8. Test Product and Placebo

The test product was pro-hydrolyzed collagen peptide without addons (CollaSel PRO^®^, 10 g Sachet, Sel Sanayi Urunleri Ticaret ve Pazarlama A.S, Istanbul, Turkey). In comparison, the placebo was 10 g of maltodextrin. Each product was administered once daily for 8 weeks.

### 2.9. Statistical Analysis

At least 102 subjects (51 for each group) were calculated to be included in this superiority study via sample size estimation based on a power of 80% at a type I error of 0.05, assuming the difference in terms of pain scoring with placebo as one and standard deviation of 2. Considering the potential dropouts, 160 subjects (80 subjects for each group) were included in this study.

Statistical analysis was performed using R Project. The independent-sample t-test was used to analyze the difference between test and placebo groups at each follow-up visit (baseline, week 1, week 4, and week 8), and the paired *t*-test was used to analyze the difference between pairs of time within a group. Data were expressed as means (standard deviation, SD), 95% confidence intervals (CIs), and percentages (%) where appropriate. *p* < 0.05 was considered statistically significant.

## 3. Results

### 3.1. Baseline Characteristics

The average age of the 160 participants (111 female, 49 male) was 52 4 ± 4.3 (Table 1).

### 3.2. WOMAC (for Knee and Hip Joints) Scores in Test Product and Placebo Groups

Except for missing data for one subject in the placebo group for the week 8 assessment, data on WOMAC scores were available in all test and placebo groups at each follow-up visit.

**Figure 2:**
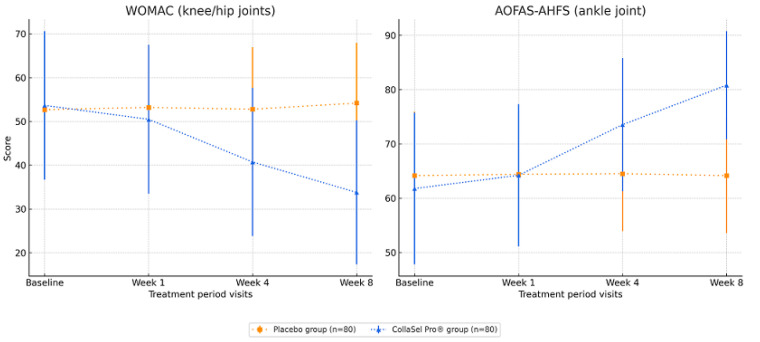
WOMAC (knee/hip joints) and AOFAS-AHFS (ankle joint) scores in the placebo and test product (CollaSel PRO^®^ hydrolyzed collagen peptide) groups.

At the initial visit, there was no significant difference in WOMAC scores between the CollaSel PRO^®^ hydrolyzed collagen peptide and placebo groups (*p* > 0.05). However, at weeks 4 and 8, the WOMAC scores were significantly lower in the collagen peptide group compared to the placebo group (all *p* < 0.001) (Table 1, Figure 2).

In the CollaSel PRO^®^ hydrolyzed collagen peptide group, the WOMAC scores at week 1, week 4, and week 8 of therapy were lower than the baseline scores (all *p* < 0.001). The significant decline in WOMAC scores was also evident throughout the treatment visits (from week 1 to week 8, *p* < 0.001 for each) (Table 2, Figure 2).

In the placebo group, no significant change was noted in WOMAC scores during the treatment period, except for the increase in mean (SD) scores from week 4 to week 8 (*p* = 0.024) (Table 2, Figure 2).

### 3.3. AOFAS-AHFS (for Ankle Joint) Scores in Test Product and Placebo Groups

Data on AOFAS-AHFS scores were available for 53 subjects in the placebo group and 45 in the test product group at each follow-up visit.

At the initial visit, the groups had no significant difference in AOFAS-AHFS scores (all *p* > 0.05). CollaSel PRO^®^ hydrolyzed collagen peptide revealed significantly higher AOFAS-AHFS score than the placebo at week 4 and week 8 (all *p* < 0.001) (Table 1, Figure 2).

In the CollaSel PRO^®^ hydrolyzed collagen peptide group, AOFAS-AHFS scores at week 1, week 4, and week 8 of therapy were significantly higher than baseline scores (all *p* < 0.001). The significant increase in AOFAS-AHFS scores was also evident throughout the treatment visits (from week 1 to week 8, *p* < 0.001 for each) (Table 2, Figure 2).

No significant change was noted in AOFAS-AHFS scores in the placebo group during the treatment period (Table 2, Figure 2).

### 3.4. Safety

A total of 46 AEs (most commonly nausea, 19.5%) were reported in 24 patients. Possible relation to test product was reported for 30 (65.2%) of 46 AEs, 97.8% of AEs were mild AEs, and no SAEs were reported. All AEs were resolved with full recovery without any action taken (Table 3).

## 4. Discussion

This randomized double-blinded, placebo-controlled, parallel trial in patients with OA-related complaints in knee, hip, and ankle joints revealed the efficacy and safety of 8-week once-daily use of hydrolyzed collagen peptide type I and type III (CollaSel PRO^®^) in OA patients. CollaSel PRO^®^ revealed significant improvement in WOMAC index (knee/hip joints) and AOFAS-AHFS scores (ankle joint) starting from the first week of the therapy. In contrast, the amelioration of pain, stiffness, and functional limitations in the knee, hip, and ankle joints continued throughout the therapy. CollaSel PRO^®^ was also superior to the placebo in ameliorating pain, stiffness, and functional limitations related to knee, hip, and ankle joints at the fourth and eighth weeks of therapy.

CollaSel PRO^®^ demonstrated clinically significant improvement in WOMAC index and AOFAS-AHFS scores, including pain, stiffness, and physical function subscales, over time (The minimal clinically important difference (MCID) thresholds ≥10–12 points for WOMAC and ≥8–10 points for AOFAS-AHFS) [23,24]. This suggests that CollaSel PRO^®^ is a valuable and effective alternative treatment in OA, with additional potential benefits in maintaining an active lifestyle and improved quality of life in OA patients throughout their aging.

While the possible beneficial effects of collagen for the treatment of OA remain controversial, several studies have reported the superiority of collagen over placebo in terms of improved WOMAC index, consistent with our results [9,11,14,25,26]. In a double-blind RCT by Benito-Ruiz et al. [27] comparing the 6-month use of oral hydrolyzed collagen (10 g once daily) vs. placebo in knee OA patients, hydrolyzed collagen was reported to be safe and effective. In another double-blind RCT by Kumar et al. [25] comparing pork skin collagen peptide (PCP, 5 g daily) or bovine bone collagen peptide (BCP, 5 g daily) vs. placebo in knee OA patients, both PCP and BCP were considered to be effective supplements for improved joint physical function and quality of life. Also, in a double-blind RCT by Mc Alindon et al. [28] comparing 6-month use of type I hydrolyzed collagen (10 g once daily) vs. placebo in knee OA patients, oral supplementation with a type I collagen hydrolysate was reported to structurally improve the thickness of articular cartilage in OA via gadolinium-enhanced MRI, suggesting potential disease-modifying capability of this agent.

Another RCT comparing hydrolyzed collagen with glucosamine sulfate in knee OA patients demonstrated that 6-month use of hydrolyzed collagen was effective and superior in improving clinical status in knee OA patients. This study reported significant improvements in pain scores, functional joint status (VAS pain scores and WOMAC index), quality of life, and favorable tolerability [29]. In addition, the use of a nutritional supplement containing hydrolyzed collagen, along with chondroitin sulfate, glucosamine sulfate, devil’s claw, and bamboo extracts, was found to be safe and effective in reducing joint pain and improving locomotor function and quality of life in patients with knee and/or hip OA, with no significant adverse events reported during the treatment [26].

Nonetheless, systemic reviews and meta-analyses revealed inconsistent findings on the efficacy of hydrolyzed collagen supplements in OA; some concluded its positive effects in ameliorating OA symptoms, whereas others reported no clinically important effects on pain and function at medium or long-term follow-ups or insufficient evidence to recommend their generalized use in daily practice for the treatment of patients with OA [9,10,12,14].

In a recent meta-analysis, all in vivo preclinical studies were concluded to reveal a potential of collagen derivatives to reduce cartilage destruction or promote cartilage repair, regardless of the type (hydrolyzed, undenatured), source (chicken, fish, porcine, bovine, squid), or molecular weight of collagen [14]. However, in vitro studies that compared different (source and size) hydrolyzed collagen preparations demonstrated a significant difference between hydrolyzed collagens from various sources in terms of their peptide composition as well as their positive or even detrimental potential effects on the OA cartilage [30,31].

Indeed, the substantial variability across the clinical trials regarding the source of hydrolyzed collagen and manufacturing processes is likely responsible for inconsistent results from clinical trials [10,14]. Moreover, given that most of the trials were of relatively short-term and small-scale studies with methodological shortcomings, extrapolating the effect from a hydrolyzed collagen to another one is considered unlikely, emphasizing the need to further address the efficacy of different hydrolyzed collagen products in OA treatment in terms of potential differences [10,14,30,32].

Given that the main structural component of the cartilage tissue is collagen type II, the use of cartilage-derived substances (type II collagen) is suggested to be a better approach for supplementing the building blocks than the use of type I and III collagen [33,34,35]. Accordingly, previous studies have reported that the use of native type II collagen has positive effects in the treatment of early osteoarthritis by increasing functional status and reducing pain [34,35]. In a double-blind RCT by Schauss et al. [33] comparing BioCell type II collagen (a hydrolyzed chicken sternal cartilage extract) vs. placebo in hips and/or knee OA patients, BioCell type II collagen was reported to be well tolerated and effective in managing OA-associated symptoms, with a significant reduction in VAS pain scores and WOMAC index, and in improving patient’s activities of daily living. In this regard, it should be noted that fourth-week WOMAC scores obtained via CollaSel PRO^®^ (type I and type III hydrolyzed collagen peptide) in our study were quite similar to the day 35 WOMAC scores (mean (SD) 40.7 (16.9) and 42.08 (12.4), respectively) reported in the BioCell type II collagen study, along with similar baseline values (53.7 (16.9) and 54.6 (11.5), respectively).

The underlying mechanism of collagen’s positive effect on OA is still not fully understood. Although mechanisms such as the stimulation of collagen biosynthesis by chondrocytes, the use of collagen-specific peptides as building blocks for articular cartilage, and the inhibition of apoptosis and hypertrophy in chondrocytes have been proposed, they have not been fully elucidated [14,36].

In a murine OA model, daily oral consumption of type I hydrolyzed collagen was reported to be chondroprotective, anti-apoptotic in the articular chondrocytes, and anti-inflammatory, providing tissue and cellular-level information explaining the evidence of its symptom relief effect in human knee OA [37]. Also, in a recent gadolinium-enhanced MRI study, type I hydrolyzed collagen was reported to affect joint structure by increasing the proteoglycan content in knee cartilage after 6 months of treatment [28], supporting the in vitro data on the stimulation of extracellular matrix synthesis by collagen peptides [38,39,40]. Additionally, it has been reported that peptides derived from hydrolyzed collagen (e.g., Pro-Hyp) increase in human blood and induce hyaluronic acid synthesis from synovial cells [39,40].

Consistently, the need for high-quality, long-term clinical studies in larger populations to confirm the chondroprotective effects of collagen derivatives and clarify their mechanisms of action is emphasized.

Considering safety, our findings align with the current evidence indicating that collagen derivatives are safe for OA patients with no significant differences between the placebo and the collagen peptide group after 6 months of treatment [9,14,25,26,32,33]. In our previous study with hydrolyzed collagen peptide CollaSel PRO^®^, no side effects were observed when 10 g of collagen peptide per day was used orally for 3 months [41].

The major strength of our study is using a pure hydrolyzed collagen peptide that is not combined with other potentially adequate nutrients and assessing OA-related joint status (pain, stiffness). However, this study has several limitations. First, the follow-up period was limited to 8 weeks, which restricts the evaluation of long-term outcomes and potential disease-modifying effects. Second, imaging or biomarker data (such as MRI, uCTX-I, uCTX-2) were not included, which could have provided further insight into structural changes. Third, knee and hip joints were analyzed together despite their biomechanical differences, which may have influenced the interpretation of treatment effects. Future studies should consider joint-specific analyses with larger sample sizes and functional limitations) in knee, hip, and ankle joints. Additionally, studies on geriatric patients are needed.

In conclusion, this study has shown that CollaSel PRO^®^ (type I and type III hydrolyzed collagen peptide) is effective in reducing OA-related pain, stiffness, and functional limitations in the knee, hip, and ankle joints, with improvements observed as early as the first week of treatment and no safety concerns. These results suggest that CollaSel PRO^®^ may serve as a safe and effective option for the symptomatic management of OA, providing pain relief and improved joint mobility. Further studies are needed to confirm any potential disease-modifying effects, potentially contributing to better quality of life as patients age. However, more significant, high-quality RCTs with diverse patient populations and extended treatment durations are needed to validate its chondroprotective effects further and clarify the mechanisms behind its clinical benefits in OA management.

## Figures and Tables

**Table 1:** Demographic and clinical characteristics of the participants for placebo and test product (Collasel PRO^®^ hydrolyzed collagen peptide) groups.

		Placebo Group (n = 80) Mean ± SD n (%)	Test Group (n = 80) Mean ± SD n (%)	*p*	95% CI
Age		52.91 ± 0.47	51.84 ± 0.48	0.149	0.143; 0.157
BMI		28.04 ± 0.39	27.71 ± 0.33	0.897	0.887; 0.899
Sex	Female	51 (63.8%)	60 (75%)	0.170	
	Male	29 (36.2%)	20 (25%)		
OA	Knee/Hip	80 (100%)	80 (100%)	0.256	
	Ankle	53 (66.3%)	45 (56.3%)		
WOMAC		n = 80	n = 80		
	Day 0	52.7 ± 15.8	53.7 ± 16.9	0.707	−6.095; 4.145
	Week 1	53.2 ± 14.2	50.5 ± 17.0	0.278	−2.196; 7.596
	Week 4	52.8 ± 14.1	40.7 ± 16.9	**<0.001**	7.180; 16.920
	Week 8	54.2 ± 13.7	33.8 ± 16.5	**<0.001**	15.637; 25.143
AOFAS-AHFS		n = 53	n = 45		
	Day 0	64.2 ± 11.8	61.8 ± 13.9	0.360	−2.772; 7.556
	Week 1	64.4 ± 11.1	64.2 ± 13.1	0.943	−4.682; 5.030
	Week 4	64.5 ± 10.6	73.6 ± 12.3	**<0.001**	−13.627; −4.465
	Week 8	64.2 ± 10.6	80.8 ± 9.9	**<0.001**	−20.801; −12.504

*p*-values < 0.05 are written in bold.

**Table 2:** WOMAC and AOFAS-AHFS scores during study visits in placebo and test product (CollaSel PRO^®^ hydrolyzed collagen peptide) groups.

	Placebo Group (n = 80)				Test Product (CollaSel Pro^®^) Group (n = 80)			
Visits	Day 0	Week 1	Week 4	Week 8	Day 0	Week 1	Week 4	Week 8
WOMAC (n)	80	80	80	79	80	80	80	80
Mean ± SD	52.7 ± 15.8	53.2 ± 14.2	52.8 ± 14.1	54.2 ± 13.7	53.7 ± 16.9	50.5 ± 17.0	40.7 ± 16.9	33.8 ± 16.5
Day 0 *p* (95% CI)		0.540 (−2.116; 1.116)	0.920 (−2.068; 1.868)	0.224 (−3.819; 0.908)		**<0.001** (1.625; 4.725)	**<0.001** (10.57; 15.28)	**<0.001** (17.027; 22.698)
Week 1 *p* (95% CI)			0.553 (−0.936; 1.736)	0.368 (−2.431; 0.912)			**<0.001** (8.175; 11.325)	**<0.001** (14.642; 18.733)
Week 4 *p* (95% CI)				**0.024** (−1.865; −0.135)				**<0.001** (5.924; 7.951)
AOFAS-AHFS (n)	**53**	**53**	**53**	**53**	**45**	**45**	**45**	**45**
Mean ± SD	64.2 ± 11.8	64.4 ± 11.1	64.5 ± 10.6	64.2 ± 10.6	61.8 ± 13.9	64.2 ± 13.1	73.6 ± 12.3	80.8 ± 9.9
Day 0 *p* (95% CI)		0.699 (−1.395; 0.943)	0.704 (−2.127; 1.447)	1.000 (−1.801; 1.801)		**<0.001** (−3.717; −1.172)	**<0.001** (−14.038; −9.518)	**<0.001** (−21.615; −16.474)
Week 1 *p* (95% CI)			0.867 (−1.466; 1.24)	0.770 (−1.318; 1.771)			**<0.001** (−11.122; −7.544)	**<0.001** (−18.718; −14.482)
Week 4 *p* (95% CI)				0.552 (−0.798; 1.477)				**<0.001** (−8.591; −5.942)

*p*-values < 0.05 are written in bold.

**Table 3:** Summary of adverse events (AEs) and safety data.

Overall Adverse Events (AEs) Summary			n (%)	
Patients with Adverse Events			24 (52.2%)	
Total Number of Adverse Events			46 (100%)	
Severity Level of AEs	Mild		45 (97.8%)	46 (100%)
	Moderate		1 (2.2%)	
	Severe		0 (0%)	
Relation to Study Drug	Certain		1 (2.2%)	46 (100%)
	Probable/likely		0 (0%)	
	Possible		30 (65.2%)	
	Unlikely		15 (32.6%)	
Type of Adverse Events	*Gastrointestinal*	Nausea	9 (19.5%)	18 (39.1%)
		Constipation	4 (8.7%)	
		Dyspepsia	2 (4.3%)	
		Stomachache	1 (2.2%)	
		Acid reflux	1 (2.2%)	
		Bloating	1 (2.2%)	
	*Skin*	Itching (hands, arms, body)	4 (8.7%)	10 (21.8%)
		Redness (arms, legs)	3 (6.5%)	
		Skin blistering on hands	1 (2.2%)	
		Dry skin	1 (2.2%)	
		Acne	1 (2.2%)	
	*Neurological*	Headache	4 (8.7%)	6 (13%)
		Tingling	1 (%)	
		Insomnia	1 (%)	
	*Other*	Joint pain	4 (8.7%)	12 (26.1%)
		Edema	2 (4,3%)	
		High blood pressure	1 (2.2%)	
		Fatigue	1 (2.2%)	
		Dry mouth and tongue with odor	1 (2.2%)	
		Dry mouth with thirst	1 (2.2%)	
		Bitterness in the mouth	1 (2.2%)	
		Weight increase	1 (2.2%)	
	*Total*			46 (100%)

## Data Availability

The datasets generated and/or analyzed during the current study are available from the corresponding author upon reasonable request.
